# Bioactive Micronutrients in Coffee: Recent Analytical Approaches for Characterization and Quantification

**DOI:** 10.1155/2014/384230

**Published:** 2014-01-22

**Authors:** Abdulmumin A. Nuhu

**Affiliations:** Department of Chemistry, Ahmadu Bello University, PMB 1069, Zaria, Kaduna 2222, Nigeria

## Abstract

Production of coffee beans is an important lifeline for the economy of several countries in Latin America, Africa, and Asia. The brew from this well sought for cash crop is readily consumed due to its good sensory qualities owing to the presence of many micronutrients. Some of these chemical compounds possess biological activities, including antiproliferative, antioxidant, and antimicrobial effects. Four representative groups of these micronutrients, namely, caffeine, chlorogenic acid, diterpenes, and trigonelline, play key roles in these bioactive effects of coffee. In order to guarantee the quality of coffee products and to protect consumer interest and safeguard their well-being, it is extremely important to employ sensitive and accurate analytical methods in the characterization and quantitative determination of these bioactive constituents. This review aims to present recent applications in this regard.

## 1. Introduction

Coffee is a popular beverage that is widely consumed around the world [1, 2]. Since its introduction into Arabia from Ethiopia many centuries ago [3], its cultivation has continued to blossom in three regions of our globe, namely, Africa, Latin America and Asia, [4–6]. Coffee is often produced from the roasted beans of a great variety of coffee crops [7]. However, *Coffea canephora* and *Coffea arabica* are the two most economically important species [8, 9]. It can now be found in both organic and conventional types [10]. Presently, the largest coffee bean producing region is Latin America [11], leading to an immense boost in the economy of the respective countries by bringing in the much needed foreign exchange. Unfortunately, market shocks, extreme weather conditions, and pests are an enormous challenge to this reality [12, 13]. World prices of coffee beans vary reasonably with their geographical source, as this affects the physical presentation of these beans and their nutritive components, two factors that are important in determination of coffee quality [14–17].

There are many nutritive substances in coffee brews which vary with the types of techniques employed in brewing processes [18]. Coffee contains mineral ingredients such as Ca, K, Fe, P, Ni, Mg, and Cr [19], polyphenols, caffeine, melanoidins, and carbohydrates [20, 21] among others. Since the variability of these chemical constituents affects price of coffee commodities by determining their qualities, there must be sensitive, precise, and accurate analytical means for their determination. It is, therefore, the aim of this paper to present a review on the recent analytical methods that have been applied to the characterization and quantification of four important bioactive constituents in coffee: caffeine, chlorogenic acid, diterpenes, and trigonelline.

## 2. Bioactive Compounds of Coffee

Coffee is rich in many bioactive substances and its consumption has been associated with many beneficial effects [22]. These include, but not limited to, reduced risk of hepatocellular carcinoma [23], antiproliferative effect against some human cancer cell lines [24], therapeutic potential against Alzheimer's disease [25], and antioxidant capacity through modulation of Nrf2 nuclear translocation [26].

### 2.1. Caffeine

**Figure 1:**
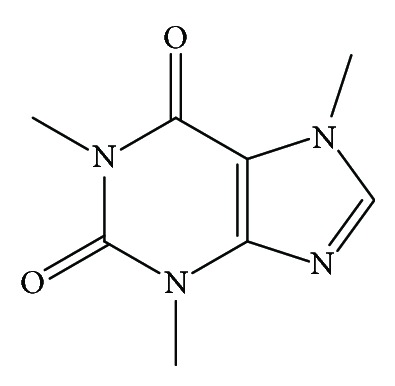
Chemical structure of caffeine.

Caffeine (C_8_H_10_N_4_O_2_), an alkaloid that is chemically known as 1,3,7-trimethylxanthine (or 1,3,7-trimethyl-1H-purine-2, 6(3H, 7H)-dione), is among the most commonly consumed stimulants worldwide [27, 28]. It contains two fused rings (Figure 1) that are related to purines [29]. Caffeine is found in many natural and processed products. Examples of these sources include green tea [30, 31], mate tea [32, 33], chocolate [34], and caffeinated beverages and energy drinks [35–37]. *C. arabica* [38] and Robusta coffee (*C. canephora* var. Robusta) [39] also contain this micronutrient.

Consumption of caffeine has shown many positive effects in various human and animal experimentations. At a dose of 6 mgKg^−1^ body mass, it was found to exhibit ergogenic effect, the ability to increase physical exercise without a concomitant increase in effort sensation, in sedentary men [40]. Its stimulatory activity was also tested, with some promising results, in persons with Parkinson's disease, where it can be used to manage both motor and nonmotor symptoms [41]. In a mouse model, crude caffeine was found to reduce the accumulation of *β*-amyloid peptides, a characteristic feature in patients suffering from Parkinson's disease [42]. Investigations have revealed that crude caffeine did possess hydrophilic antioxidant activity (145 *μ*mol Trolox equivalent (TE)/g) and lipophilic antioxidant activity (66 *μ*mol TE/g), and its administration has led to the inhibition of cyclooxygenase-2 enzyme better than aspirin [43].

Some of the adverse effects of high doses of caffeine include decrease tonus of the lower esophageal sphincter muscle, overstimulation of central nervous system, intrauterine growth retardation, and higher risk of miscarriage [44]. Withdrawal from caffeine ingestion may present with lower mental alertness, diminished performance, and over sleepiness [45].

### 2.2. Chlorogenic Acid

**Figure 2:**
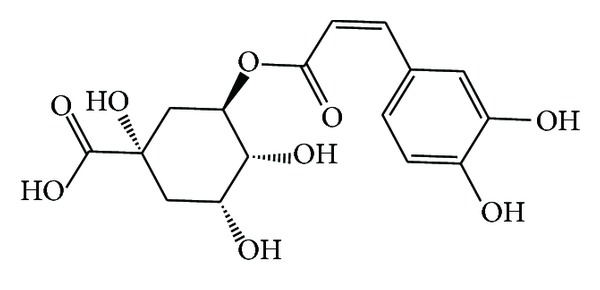
Chemical structure of chlorogenic acid.

Chlorogenic acid (CGA) (Figure 2), which has nothing to do with chlorine, is an ester that is formed from the reaction of caffeic acid with L-quinic acid; hence the name 5-caffeoylquinic acid (5-CQA) [46, 47]. It is a polyphenolic compound that is abundant in many plants [48]. It is present in tobacco leaves [49], mulberry tree [50], and coffee beans [51]. It is found to be responsible for the astringent taste of coffee brews [52, 53]. Many positive effects have been attributed to CGA. For instance, its hypolipidemic effect has led to weight reduction in experimental mice [54, 55]. The antioxidant activities of this phytochemical were exhibited against ischemia-reperfusion injury [56] and in the protection against oxidative damage of macromolecules such as DNA, lipids, and proteins [57]. When administered to mice under scopolamine-induced amnesia, CGA has shown neuroprotective function via the inhibition of acetyl cholinesterase [58].

Different microbial entities are susceptible to CGA's antimicrobial activities. With respect to viruses, CGA has shown antihepatitis B virus potency in a duckling model [59] and potential anti-H1N1 influenza virus [60]. CGA analogs that showed appreciable anti-HIV activities have been synthesized [61]. CGA is not only active against viral particles, but it has also antibacterial and antifungal functions. At minimum inhibitory concentrations (MIC) of 20–80 *μ*g/mL, the growth of all bacterial pathogens tested was inhibited by CGA [62]. It is argued that the high resistance of immature peach fruits to the brown rot fungus, *Monilinia laxa*, is due to their high contents of CGA [63].

### 2.3. Diterpenes

Diterpenes are a group of terpenoids commonly found as secondary metabolites in terrestrial and marine organisms [64]. They often occur in a C-20 backbone where isoprene units combine in different forms to give an array of diterpenes such as abietane [65], cembrane [66], guanacastepene A [67], quinonoid [68], jatropha [69], and cafestol and kahweol [70] types. These and several others may be found in coffee [71], tea [72], and *Salvia *spp. [73]. Some types have been isolated from the fungus *Trichoderma longibrachiatum *[74] and from sponge family [75].

**Figure 3:**
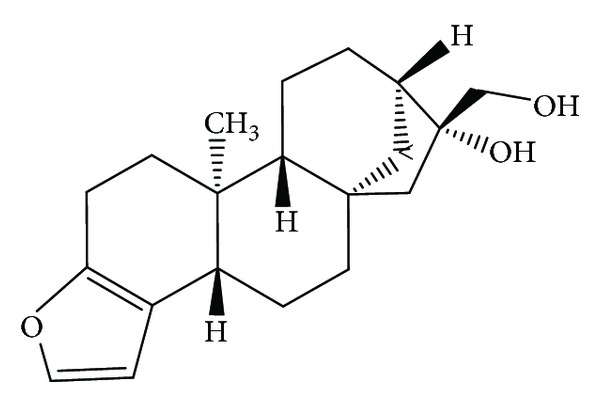
Chemical structure of the diterpene cafestol.

Diterpenes have displayed several types of bioactivities via different mechanisms. A diterpene alkaloid isolated from the Caribbean sponge *Agelas citrina* has shown antifungal function [76]. Experiment with oridonin type has demonstrated its antiproliferative effect against leukemia-derived Jurkat cells [77]. Abietane type was cytotoxic to human pancreatic cancer cell line MIA PaCa-2 [78]. Antioxidant function is found in *Salvia officinalis* [79], and an isolate of the Brazilian alga *Dictyota menstrualis* has indicated anti-HIV potential of this phytochemical [80]. Antimicrobial activities are found in a recent review [81]. Some diterpenes are toxic to insects [82] and to embryos of medaka, Japanese rice fish [83]. Structure of cafestol is given in Figure 3.

### 2.4. Trigonelline

**Figure 4:**
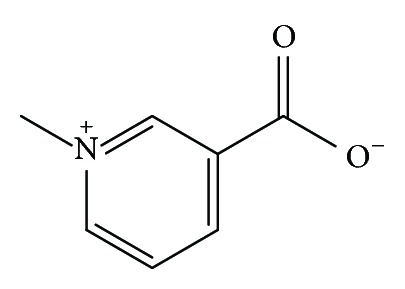
Chemical structure of trigonelline.

Trigonelline (TRG) (Figure 4) is a nitrogenous compound, a pyridine alkaloid that is derived from the methylation of the nitrogen atom of nicotinic acid (niacin); hence the name N-methyl nicotinic acid [84–86]. TRG is found in pumpkin seeds [87], mangrove legumes [88], *Moringa oleifera* tree [89], and coffee [90, 91]. It is used as roasting level discriminator in both Arabica and Robusta coffees [92].

TRG has a proven antidiabetic effect; its administration to model rats suffering from diabetes mellitus has resulted in reduced blood glucose levels in oral glucose tolerance test [93]. It has also shown beneficial effects in rats displaying peripheral neuropathy, a condition for which there is no effective drug for its treatment [94]. Its function as an inhibitor of Nrf2 gene transcription has caused pancreatic cancer cells to be more susceptible to cell death through apoptosis [95]. At an MIC of 0.8 mg/mL, TRG content of *C. canephora* extracts has correlated positively with reduction in biofilm formation via bacteriostatic action on *Streptococcus mutans* [96].

## 3. Extraction Methods for Bioactive Micronutrients of Coffee

Extraction and preconcentration procedures may be necessary for any good and sensitive characterization and determination of analytes, especially when these are present in very low concentrations. Many traditional and conventional methods have been employed in the extraction of bioactive micronutrients of coffee preparatory to their analysis.

### 3.1. Solid-Liquid Extraction

Different types of coffee brewing techniques have profound influence on the types and amounts of chemical compounds in coffee [18]. These techniques, all of which are based on solid-liquid extraction (SLE), include Boiled, Morcha Pot, French press, Turkish, Plunger, and Espresso types [97, 98]. On comparison, Turkish style and French press provided the highest amounts of kahweol and cafestol compared to others [71]. In SLE, water is often the solvent for extraction, which is beneficial to polar and water-soluble analytes [70]. For analytical determinations, extraction can be carried out in other solvents but may be reconstituted in water before further analysis [89]. Bi et al. [99], however, found that a mixture of ethanol and water (50/50, v/v) was most effective for removal of caffeine from coffee waste. During a 90 min extraction, 60% methanol and a solvent/solid ratio of 40 mL/g spent coffee grounds provided the best conditions for obtaining extract with high phenolic compounds, including CGA [100]. Because the main discriminator in these SLE methods is polarity, it leads to limited selectivity in extraction; when caffeine was analyzed in roasted coffee ground, not only caffeine was extracted with the SLE procedure [101]. This method of extraction may, therefore, present interference problems during instrumental analysis.

Other conditions that define the performance of SLE procedures are the effect of temperature and duration of extraction [92, 102]. As a result of different temperature sensitivities of the complex mixture of constituents in coffee, it is difficult to satisfy the temperature needs of individual analytes in a single-extraction system. In an attempt to eliminate this challenge, a double-extraction method was described which accommodated the extraction of both thermolabile CGA and thermostable caffeine, with high recoveries [20]. In this system, one batch is fed into selected stages at mild temperature and pressure while another is fed into remaining stages at higher temperature and pressure, and 50% of extracts from each stream are mixed and lyophilized. Apart from being cumbersome, multistep SLE may lead to loss of some analytes. It was recently found that proprietary ethanol single-step extraction resulted in higher content of caffeine than multistep procedures [103].

### 3.2. Liquid-Liquid Extraction and Solid-Phase Extraction

Liquid-liquid extraction (LLE) is one of the most widely used traditional sample preparation techniques [104]. The disparity in solubility of an analyte in two immiscible solvents is harnessed for its extraction. For the extraction of the four bioactive constituents of coffee, LLE can be practiced by extracting these analytes from aqueous medium to an organic one. However, because each of these constituents has, at least, a moderate solubility in water, application of LLE in this regard is limited. In one example, caffeine was extracted from water into dichloromethane, while CGA remained in the residual aqueous solution [105]. To solubilize a polar analyte in an organic solvent during an LLE procedure, especially when gas chromatographic analysis is intended, it is sometimes necessary to add ionic surfactants in the extraction medium [106, 107].

Solid-phase extraction (SPE) utilizes the sorptive capacity of a stationary sorbent in SPE cartridge in order to extract analytes. It starts by conditioning the cartridge, after which the sample is run through, then dried, and finally eluted using a suitable solvent [108, 109]. Recently, Caprioli et al. [110] have explained the application of SPE for the extraction of CGA in Arabica and Robusta coffees using Strata-X cartridge. Low temperatures and methanol as extraction solvent resulted in the most efficient extraction of CGA.

Both LLE and SPE applications are dwarfed by being multistep and environmentally unfriendly due to large solvent volumes involved, especially with regard to LLE.

### 3.3. Microwave-Assisted and Ultrasound-Assisted Extractions

Microwave-assisted extraction (MAE) is a solvent reduction extraction method that uses electromagnetic energy to assist (enhance) extraction of analytes. Microwave interacts with matter in a special way that causes instantaneous heating, thus facilitating desorption of analytes from their matrices and improving mass transfer rate [111, 112]. Compared to conventional solvent extraction, MAE is faster and involves minimal use of solvent. In just under 40 s, maximum phenolic content was obtained from spent filter coffee under the influence of low concentrated ethanol [113]. Hongcheng et al. [114] have developed a fast MAE method for the simultaneous extraction of TRG, caffeine and nicotinic acid from coffee using a pressure of 200 psi. Other conditions that can affect this procedure include temperature and wattage. At the temperature of 50°C and wattage of 800 w, CGA- and caffeine-rich extracts were prepared from coffee beans [115].

Another assisted extraction procedure is ultrasound-assisted extraction (UAE), a procedure that uses sound frequency to agitate extraction medium and speed up contact between solvent and matrix. It has found a number of applications in the extraction of phytochemicals [50, 116]. The energy burst from UAE equipment causes thermal heating that reduces extraction time. Wang et al. [117] have found that quantity of caffeine extracted from coffee reached saturation in only 15 s and augmented positively with increase in temperature at an operating frequency of 28 kHz. UAE was successfully applied to the determination of TRG in coffee powder and instant coffee with excellent recoveries [118].

Some of the demerits of MAE and UAE include the difficulty in selecting appropriate extraction conditions (including type of solvent, pressure, and frequency) that would not cause excessive heating and lead to degradation of some analytes, necessity of clean-up after extraction, and relative high cost of equipment.

### 3.4. Other Extraction Methods

The combination of gas-like mass transfer and liquid-like solvating abilities of supercritical fluids is exploited in the extraction method known as supercritical fluid extraction (SFE). In this method, a solvent at its supercritical state is used to speed up extraction by an increased matrix-penetrating ability and enhanced mass transfer potential. Although large industrial application of SFE is often hindered by the trade-off between selectivity and recovery efficiency, this method is still found in routine laboratory applications [79, 119]. Among the numerous factors that affect performance of this approach, temperature and pressure are key considerations in recovery and selectivity calculations. Machmudah et al. [120] have found that recovery of caffeine and the separation factor of CGA from caffeine were positively correlated with increasing height (pressure) of nonpolar recovery section in the separation system using supercritical carbon dioxide (SCCO_2_) in water. They also found that recovery of caffeine in the SCCO_2_ phase increased with decreased temperature. This could only be true at pressures below the crossover pressure. Above the crossover pressure (~200 bar), however, an increase in temperature resulted in an increased recovery as observed in the extraction of caffeine from Robusta coffee husks [39].

Solid-phase microextraction (SPME) was introduced in the 1990s in response to the challenges posed by SPE. It basically involves sorption of an analyte to a commercial fiber coated with polymeric substance, followed by a desorption step using a thermal process or suitable solvent. SPME allows for solvent minimization and provides for higher sample enrichment. Different forms of SPME techniques are practiced, but the headspace type (HS-SPME) is very suitable in the analysis of volatile compounds [121, 122]. Affinity of an analyte towards the fiber can be enhanced by carefully selecting the right polymer coating material [123]. López-Darias et al. [124] found that poly-(ViHIm^+^Cl^−^) gave excellent performance for aromatic alcohols while polyacrylamide coating performed better for heterocyclic aromatics. Temperature and extraction time also influence the performance of this method as noticed in a different experiment where poly-dimethyl-siloxane in combination with divinylbenzene (PDMS/DVB) SPME fiber with a 65 *μ*m thick film coating was used for the extraction of caffeine and CGA in coffee [125]. Some of the shortcomings of SPME include leaching of coating material, breaking of fiber, and bending of the syringe support.

## 4. Methods for Characterization and Quantification of Bioactive Micronutrients of Coffee

Due to the economic importance of coffee and its constituent bioactive substances, consumer interest and safety should remain paramount. Therefore, there is an increasing demand for proper quality control for certification of contents and reducing counterfeits and substandard products. To achieve these noble goals, sensitive and accurate analytical methods for both qualitative and quantitative determinations and characterization of chemical substances in coffee are required. There are many analytical procedures (Table 1), including chromatographic, photometric, spectral, and electrochemical methods that are geared toward this end [126, 127].

### 4.1. Separation-Based Methods

To reduce interference and increase sensitivity, an extract is commonly run through a separation system, followed by detection of separated contents using a suitable detector. Among the many analytical techniques that have been applied in the determination of coffee constituents, high performance liquid chromatography (HPLC), gas chromatography (GC), and capillary electrophoresis (CE), in combination with various detectors, such as ultraviolet/visible (UV/Vis) detector, diode array detector (DAD), refractive index (RI) detector, and chemiluminescence detector, are often applied for the determination of nonvolatile, volatile, polar, and nonpolar classes of analytes.

#### 4.1.1. HPLC Methods

This is one of the most powerful tools available for analytical applications in many fields. Because of its versatility, low limits of detection (LOD), and accessibility, its applications can now be found in numerous methods for the analyses of industrial chemicals, agroallied chemicals, and constituents of foods and beverages. It is the single most applied separation-based method of quantification for bioactive constituents in coffee and coffee products. A HPLC-based method for TRG determination and for the quality control of coffee products was explained by Liu et al. [118]. After its separation, TRG was detected at 260 nm *λ*. The total contents of CGA and its isomers in a cup of certified Italian Espresso coffee were between 1522.5 and 2223.4 mgKg^−1^ as determined by HPLC-DAD [110]. Results of HPLC-UV/RI analysis of 17 Brazilian Arabica cultivars have revealed that semidry processing method showed lower levels of CGA and TRG than coffee processed by wet method, which goes to confirm the effect of different processing methods on constituents of coffee [128]. In another experiment, HPLC-UV/Vis analysis revealed that coffee contains 0.06–2.55% caffeine and this value decreases with roasting intensity [129].

HPLC can be performed in either reverse or normal phase mode. A caffeine assessment tool (CAT) was developed with results obtained from a reverse-phase- (RP-) HPLC-UV analysis of several coffee and coffee-based beverages [130]. Moreira and Scarminio [131] have applied RP-HPLC-DAD for the determination of caffeine at 275 nm *λ* using a binary ethanol-dichloromethane solution (1 : 1) as mobile phase. Mobile phase in gradient flow mode is often employed to resolve complex mixtures. When different coffee extraction methods were tested using a mixture of 0.1% formic acid in water and 0.1% formic acid in acetonitrile in this type of flow mode, CGA and caffeine contents of the different extracts were different, confirming that contents may vary according to method of extraction [125].

Two-dimensional HPLC (2D-HPLC) may provide for better resolution than in ordinary practice. The application of this system in combination with chemiluminescence detector allowed for increased sensitivity and better selectivity in the determination of antioxidant constituents of three Espresso coffees (“Ristretto,” “Decaffeinato,” and “Volluto”) [132]. Elsewhere, a C-18 and Bondapak NH_2_ columns in series were used for the simultaneous determination of TRG, caffeine, and nicotinic acid [114]. This method selected conditions optimal at 120°C and 200 psi, and good separation was achieved in just 3 min, driven by 0.02 M phosphoric acid-methanol (70 : 30, v/v) mobile phase at 0.8 mL/min.

Apart from the common detection systems (UV/Vis, DAD, and RI) often employed in liquid chromatographic applications for the characterization of these coffee contents, applications of MS detection are also found in the literature. By a careful selection of parameters, molecular markers for distinguishing between different coffee samples or between constituents can be assigned [133]. In a method of liquid chromatography/multistage spectrometry (LC/MS^*n*^) (*n* = 2–4) described by Jaiswal and Kuhnert [134], hitherto unreported isomers of CGA (triacyl CGAs) were detected in Robusta coffee bean extracts and their structures assigned using LC/MS^*n*^ fragmentation patterns. In combination with similar fragmentation patterns (LC/MS^*n*^, *n* = 2-3), relative hydrophobicity and fragmentation analogy were also harnessed to distinguish between two isomeric classes of CGA (CGA lactones and cinnamoylshikimate) which are formed from CGA by loss of water at high processing temperatures [135]. Interestingly, a high temperature reverse phase liquid chromatography coupled with isotope ratio mass spectrometry (HT-RPLC/IRMS) was employed to distinguish between natural and synthetic caffeine in coffee drinks, without the painful need for extraction, by using *δ* ^13^ C values (between 25 and 32‰ for natural caffeine, and between 33 and 38‰ for synthetic caffeine) as distinguishable groups [136]. This method will be a special aid in the fight against counterfeiting and adulteration of coffee products.

#### 4.1.2. CE and Other Methods

CE uses a very small diameter bore capillary to effect separation of charged analytes dissolved in a suitable buffer. The driving force for this separation system is the use of high voltage that influences electrokinetic migration of analytes to either of the positive and negative poles. Hence, a candidate analyte for CE must possess a net negative or positive charge for effective separation. In the last five years, CE has been applied in many fields including the analysis of plant materials [86]. While HPLC did provide lower values of LOD for the determination of caffeine in decaffeinated coffee than CE, the latter was 30% faster, had lower cost of reagents, and generated smaller volumes of residues [137]. In a similar experiment, a fast hydrodynamic injection (50 mBar, 7 s) was applied in the analysis of caffeine in commercial decaffeinated coffee using a 48 cm × 50 *μ*m fused-silica capillary [138]. Other optimized conditions for this method include a mixture of sodium carbonate (10 mmolL^−1^) and sodium dodecyl sulfate (50 mmolL^−1^) as buffer, 15 kV voltage, and 206 nm *λ*. Elsewhere, Li et al. [139] have explained a CE method for the determination of caffeine in roasted coffee. This method is characterized by extremely high precision; percent relative standard deviation (%RSD) was less than 0.8% for retention time and 1.7% for peak areas.

GC determination can be extremely important, especially in the analysis of volatile components of coffee [108, 121]. For semivolatile and nonvolatile portions, however, alternative methods will be more desirable and effective. One of such methods is based countercurrent chromatography (CCC), which differs significantly from the preceding types of separation systems by not involving any solid support. Instead, two immiscible solvents are used, one solvent apiece for the stationary and mobile phases. This is exemplified in the method of Machmudah et al. [120] where SCCO_2_ and water were used, in countercurrent flow mode, to effect the separation of CGA from caffeine. Recently, a method based on the high-speed mode (HSCCC), which could be used for the preparative isolation of milligram amount of CGA from coffee, was explained [140].

### 4.2. Separation-Less Methods

In many instances, determination of analytes is performed without the need to go through a separation step. Molecular behaviors, such as mass fragmentation patterns, magnetic resonance, electrical properties, and UV/Vis or IR absorbance, are used for their direct characterization and/or quantification. These methods can be defined by extremely low LODs, high throughput, and good selectivity and sensitivity in their applications to the analysis of coffee micronutrients.

#### 4.2.1. Spectroscopic Methods

These methods are based on light-absorbing or light-emitting properties of molecules as they interact with electromagnetic radiation. As a molecule absorbs light energy of defined wavelength, electronic transition characteristic of that molecule is elicited, and this can be used for the characterization and quantification of such molecule. At 324 nm, CGA has a strong *π*-π* transition resulting from the delocalized electrons on benzene ring, and this was used for its characterization and quantification in coffee bean extract [105]. Null hypothesis was obeyed between the results obtained by this method and results of HPLC analysis.

Many determinations of antioxidant activities in coffee by different mechanisms are based on UV/Vis absorption spectroscopy [113]. CGA and caffeine are among the main antioxidant micronutrients in coffee. Their antioxidant capacities were measured using 2,2′-azino-bis(3-ethylbenzothiazoline-6-sulphonic acid (ABTS), 2,2-diphenyl-1-picrylhydrazyl (DPPH), and Folin-Ciocalteau [102]. In a similar investigation, antioxidant capacities of these hydrophilic compounds were also investigated using disodium nitrosodisulfonate (Frémy's salt) and 2,2,6,6-tetramethyl-1-piperidinyloxy (TEMPO), and results for different coffee brewing techniques were obtained as 46.0–102.3% (Filter), <42% (Plunger), and 85.6% (Espresso) [98]. DPPH-radical scavenging activity greater than 75% was recorded in the extract of green coffee beans containing 22–40% caffeine and 31–62 CGA [115].

#### 4.2.2. Electrochemical Methods

Oxidation-reduction behaviors of analytes at electrode surfaces may be used for the characterization of such analytes based on their specific relationships with potential and current, or other electrical quantities. Different types of voltammetry and coulometry have been applied in the determination of coffee constituents with high sensitivity, selectivity, and speed. For instance, oxidation of CGA at the surface of boron-doped diamond electrode, using adsorptive transfer stripping voltammetric conditions, was applied for the electrochemical estimation of antioxidant properties of coffee samples with very low LOD (49 ng/mL) and good linearity within the concentration range of 0.25–4.00 *μ*g/mL [141]. Direct determination of caffeine was achieved by square-wave voltammetry (SWV) using a highly selective pseudo carbon paste electrode versus SCE [142].

Different additives may influence the performance of these methods in several ways. While milk proteins (casein and bovine serum albumin) formed complexes with polyphenolic constituents in coffee thereby significantly reducing their available OH groups, and thus their antioxidant capacities, as revealed by coulometric titration with hexacyanoferrate (III) ions [143]; addition of cationic surfactant (cetyltrimethylammonium bromide) in the supporting electrolyte (0.04 mol L^−1^ phosphate buffer, pH 6.0) significantly enhanced the reduction of caffeine at hanging mercury drop electrode, determined by both differential-pulse polarography (DPP) and SWV [144]. When the surface of a glassy carbon electrode was modified by the deposition of thin film of 4-amino-3-hydroxynaphthalene sulfonic acid (AHNSA), highly selective electrocatalyzed oxidation of caffeine was achieved leading to its high recoveries in coffee extracts [145].

Recently, an electrochemical sensor for the selective determination of CGA in coffee samples by differential pulse voltammetry (DPV) was constructed from the deposition of a thin film of molecularly imprinted siloxane (MIS) on gold surface [146]. In a similar application by Fernandes et al. [147], CGA was selectively determined by SWV at +0.25 V (versus Ag/AgCl) using a biosensor constructed from 1-n-butyl-3-methylimidazolium hexafluorophosphate containing dispersed iridium nanoparticles (Ir-BMI.PF_6_) and polyphenol oxidase.

#### 4.2.3. Spectral Methods

Analytical characterization and quantification can also be achieved through stand-alone spectral revelations. MS fragmentation patterns, under different ionization modes, have been employed in chemical analysis, including analysis of components of coffee [148]. Direct analysis in real time (DART) coupled to time-of-flight mass spectrometry (TOFMS) yielded a high throughput (<1 min per run) analysis of caffeine in coffee samples [101]. Without the need for sample preparation or chromatographic separation, the molecular ion (M + H)^+^ resulting from the method of DART-MS was used for the identification of caffeine in commercial instant coffee samples [149]. Caffeine has a mass to charge ratio (*m*/*z*) of 194, and its characterization alongside kahweol (*m*/*z*, 296) and cafestol (*m*/*z*, 298), two marker compounds for *C. arabica* and *C. canephora*, was achieved in the method of single photon ionization coupled with TOFMS (SPI-TOFMS) developed by Hertz-Schünemann et al. [150] for the recognition of roasting gas profiles of coffee.

Characterization of bioactive components of coffee can also be achieved by using their near-IR (NIR) (2500–4000 cm^−1^) absorption spectra. Methods based on this approach are nondestructive and can offer reliable speed of determination. In addition to qualitative analysis, chemometric models can assist in quantitative interpretation of NIR spectral data. In one example, caffeine, TRG, and CGA were determined as components of sensory quality in Arabica roasted coffee samples using ordered prediction selection (OPS) algorithm, partial least squares (PLS), and NIR spectra [151]. Recently, Zhang et al. [152] developed an improved regression model for NIR determination of caffeine. This method is characterized by root mean square error of cross validation (RMSECV) of 0.375 mg/g, %RSD of 1.707%, and correlation coefficient of 0.918 at PLS factor of 7.

For some time now, nuclear magnetic resonance (NMR) spectrometry has been offering good information for the identification and structural elucidation of compounds, and this has now been extended to coffee constituents. Using ^1^H-NMR and partial least squares discriminant analysis (OPLS-DA) models, TRG and CGA were found among the main metabolites that can characterize and separate *C. arabica* samples according to their geographical origin [153]. Recently, structures of CGA lactones, which hitherto were not available in the literature, were established from NMR data [140]. Self-association and complexation of coffee constituents, both in coffee brews and in aqueous media, can pose serious challenge for their analytical resolutions. D'Amelio et al. [154] have investigated structural features of CGA-caffeine complexation using high resolution ^1^H-NMR. NMR can also offer quantitative information on chemical components in coffee. Without a nuclear Overhauser effect, three isomers of CGA were identified and quantified in green coffee bean extract through integration of 2D NMR carbon signals by use of relaxation reagent and inverse-gated decoupling method [155]. According to del Campo et al. [156], caffeine and TRG, among other constituents of coffee, can be quantitatively analyzed by use of their singlet ^1^H-NMR signals within 7.6–9.5 ppm and the use of 3-(trimethylsilyl)-2,2,3,3-tetradeuteropropionic acid both as internal standard and as reference for *δ* = 0.00 ppm.

## 5. Conclusions

Coffee is a popular beverage that is obtained from different varieties of coffee beans. This important drink contains many micronutrients, some of which have proven bioactivities, such as anticancer, antimicrobial, antioxidant, and host of other effects. Four important groups of these biologically important micronutrients, namely, caffeine, CGA, diterpenes, and TRG, have been discussed in this review. Because of their importance in biological systems, recent analytical methods for their characterization and quantitative determination have also been reviewed. It is envisaged that proper applications of these methods will go a long way in aiding quality assurance and safeguarding consumer safety and satisfaction.

## Figures and Tables

**Table 1:** Analytical methods for characterization and quantification of bioactive micronutrients in coffee.

Micronutrient	Method	Figure of merit	Reference
CGA	SWV	LOD (0.049 *μ*g mL^−1^)	Yardim, 2012 [141]
CGA, caffeine	UV-Vis	%R^a^ (91–98.43%), CV^b^ (2.80–5.45%)	Belay and Gholap, 2009 [105]
TRG	HPLC	%R (>90%), %RSD (<3%)	Liu et al., 2011 [118]
Caffeine	NIR/chemometrics	%RSD (1.707%), RMSEP (0.378 mg/g)	Zhang et al., 2013 [152]
TRG, caffeine	HPLC	LOD (0.02 mg/L), LOQ (0.005%)	Hongcheng et al., 2012 [114]
Caffeine	DART-TOFMS	%R (97–107%), %RSD (<5%)	Danhelova et al., 2012 [101]
TRG, caffeine	^ 1^H-NMR	CV (2.4, 4.2%), LOD (0.58, 1.32 mg/g)	del Campo et al., 2010 [156]
CGA	DPP, SWV	LOD (0.236 *μ*molL^−1^, 1.34 nmolL^−1^)	Araújo et al., 2009 [144]
CGA isomers	2D-NMR	%R (75–80%), R^2^ (0.9960–0.9994)	Wei et al., 2010 [155]
Caffeine	CE, HPLC	LOD (0.87, 0.021 mg/100 g), %RSD (<2%)	Bizzotto et al., 2013 [137]
Caffeine	CV^c^	LOD (0.348 *μ*molL^−1^), R^2^ (0.999)	Mersal, 2012 [142]
Caffeine	CE	%RSD (<2%)	Li et al., 2009 [139]
CGA	DPV	LOD (0.148 *μ*molL^−1^)	Santos et al., 2011 [146]
CGA isomers	HPLC	%R (67–99%), R^2^ (>0.99)	Caprioli et al., 2013 [110]
CGA	Biosensor/SWV	LOD (0.915 *μ*molL^−1^), %R (93.2–105.7%)	Fernandes et al., 2009 [147]
Cafestol, kahweol	HPLC	%RSD (<5%)	Sridevi et al., 2011 [71]
Caffeine	CV/SWV	LOD (0.137 *μ*molL^−1^), %R (>90%)	Amare and Admassie, 2012 [145]

^a^Percent recovery, ^b^coefficient of variation, ^c^cyclic voltammetry.
